# Incomplete Endothelialization, at 2 Years and 8 Months Postimplantation, of a WATCHMAN Left Atrial Appendage Closure Device

**DOI:** 10.1016/j.cjco.2026.02.018

**Published:** 2026-02-28

**Authors:** Satoko Ryuzaki, Yusuke Kondo, Takanori Aihara, Jun-ichiro Ikeda, Hironobu Nishiori, Goro Matsumiya, Yoshio Kobayashi

**Affiliations:** a Department of Advanced Arrhythmia Bioengineering, Chiba University Graduate School of Medicine, Chiba, Japan; b Department of Cardiovascular Medicine, Chiba University Graduate School of Medicine, Chiba, Japan; c Department of Pathology, Chiba University Hospital, Chiba, Japan; d Department of Diagnostic Pathology, Chiba University Graduate School of Medicine, Chiba, Japan; e Department of Cardiovascular Surgery, Chiba University Graduate School of Medicine, Chiba, Japan

**Keywords:** WATCHMAN, endothelialization, left atrial appendage closure, left atrial appendage occlusion

A 69-year-old man with dilated cardiomyopathy–related heart failure underwent implantation of a cardiac resynchronization therapy defibrillator (CRTD). Because long-term anticoagulation for persistent atrial fibrillation caused gastrointestinal bleeding, he subsequently underwent percutaneous left atrial appendage closure (LAAC) with a WATCHMAN 2.5 device (Boston Scientific, Marlborough, MA). At the initial implantation, the device fulfilled the PASS criteria (Position, Anchor, Size, and Seal), without excessive compression, oversizing, or a prominent shoulder. As the postprocedure antithrombotic therapy, edoxaban 15 mg/d was initiated after LAAC; however, it was discontinued 2 months after implantation because of recurrent gastrointestinal bleeding, with a hemoglobin nadir of 5.5 g/dL requiring transfusion. Antithrombotic therapy was withheld for 1 month and subsequently switched to clopidogrel 75 mg/d, which was continued until device explantation. No device-related thrombus or significant peri-device leak was observed during follow-up care.

Two years and 8 months later, due to heart failure, severe mitral regurgitation, and CRTD infection, he underwent mitral valve repair and simultaneous removal of the infected CRTD system with prophylactic removal of the Watchman device, given that device infection could not be excluded, although inadequate endothelialization was not suspected preoperatively.

Angioscopy was performed intraoperatively after device explantation to directly assess endothelial coverage of the device surface. Gross inspection at the time of explantation, followed by intraoperative angioscopy, revealed incomplete endothelial coverage of the device surface (Fig. 1). Histologic evaluation showed no fibrous tissue, no inflammatory cell infiltration, and no bacteria on Gram staining (Fig. 2).

**Figure 1:**
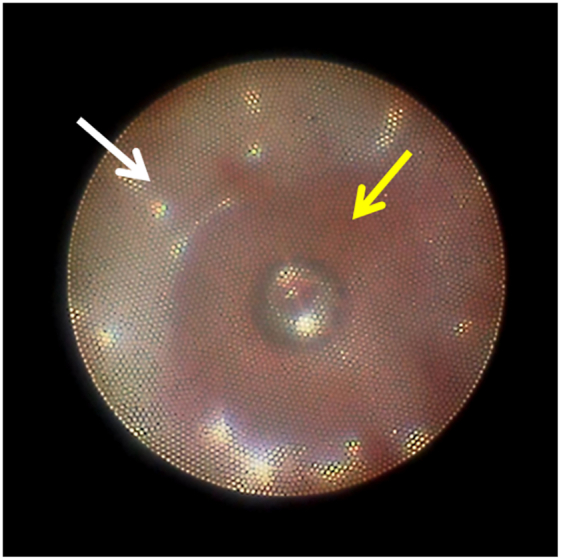
Angioscopic findings of the explanted WATCHMAN device. The image focuses on the hub region. Endothelialized areas are indicated by a **white arrow**, and nonendothelialized areas are indicated by a **yellow arrow**.

**Figure 2:**
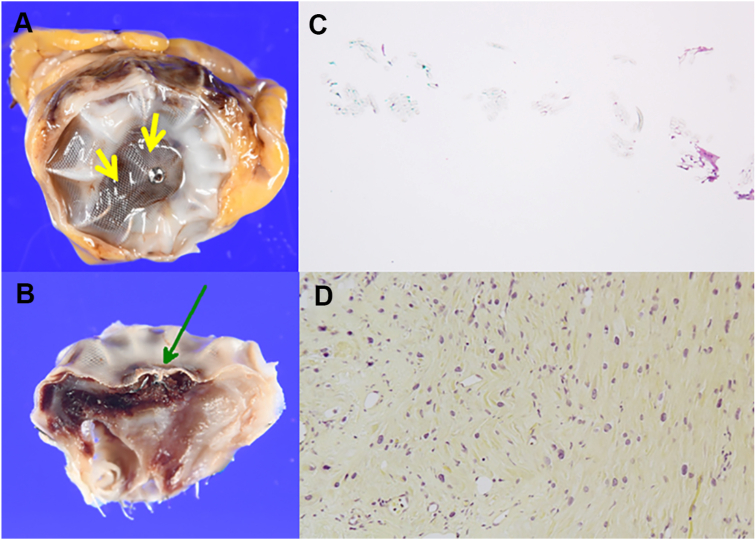
Pathologic observation of (**A**) the resected WATCHMAN device. Nonendothelialized areas around the hub are indicated by **yellow arrows**. (**B**) Incomplete endothelial coverage was observed at the central portion of the device. The hub is marked by a **green arrow**. (**C**) Microscopic examination using hematoxylin and eosin staining. The green-stained area represents the region closest to the attachment hub. Bare device components with exposed fabric and exposed attachment hubs were observed, without identifiable cellular components, indicating absence of endothelialization in the central portion of the device. (**D**) Gram staining revealed no microorganisms, with no findings suggestive of bacterial infection.

Previous studies have reported complete endothelialization of the WATCHMAN device by 9 months in humans and within 1 month in most canine models.^1^^,^^2^ Recent contrast-enhanced computed tomography studies have reported that transfabric leaks (TFLs) are observed in 27%-52% of patients at approximately 4 months after left atrial appendage occlusion, suggesting that device endothelialization in humans may not necessarily follow the time course demonstrated in animal models.^3^ This case is the first to directly demonstrate incomplete endothelialization 2 years and 8 months after implantation, highlighting the need for individualized post-LAAC management and further investigation into long-term device healing. Previous reports have demonstrated an association between transfabric leaks and both a device size ≥ 27 mm and a CHA_2_DS_2_-VASc score (congestive heart failure, hypertension, age ≥75 years [2 points], diabetes mellitus, stroke/transient ischemic attack [2 points], vascular disease, age 65–74 years, and sex category [female]) ≥ 4; notably, the present case exhibited both risk factors, with a 30-mm device and a CHA_2_DS_2_-VASc score of 4.^4^ Newer-generation devices, such as the WATCHMAN FLX Pro (Boston Scientific, Marlborough, MA), which incorporates an antithrombotic surface coating designed to promote earlier and more complete endothelialization, may represent a future alternative to address this limitation; however, long-term clinical data are still warranted.^5^

**Novel Teaching Points**

• Complete endothelialization of LAAC devices cannot be assumed, even several years after implantation, and delayed or incomplete healing may occur in selected patients.
• The absence of device-related thrombus or significant peri-device leak on follow-up imaging does not necessarily indicate complete endothelial coverage of the device surface.
• Intraoperative angioscopy and histologic examination at the time of device explantation provide a unique opportunity to directly evaluate device healing in humans.
• Although newer-generation LAAC devices incorporate surface modifications to promote endothelialization, robust long-term clinical data are still required.

## Ethics Statement

This study was conducted in accordance with the Declaration of Helsinki.

## Patient Consent

The authors confirm that patent consent forms have been obtained for this article.

## Funding Sources

The authors have no funding sources to declare.

## Disclosures

Y. Kondo reports receiving lecture fees from Daiichi-Sankyo, Medtronic, Abbott Medical Japan, Biotronik Japan, Boston Scientific, and Japan Lifeline; research funds from Daiichi-Sankyo and Boston Scientific; and proctoring fees from Boston Scientific, outside the submitted work. Y. Kobayashi reports receiving lecture fees from Abbott Medical Japan, Bayer Japan, Bristol-Myers Squibb, Boehringer Ingelheim, and Daiichi-Sankyo; and scholarship funds from Takeda Pharmaceutical, Abbott Medical Japan, Terumo, Otsuka Pharmaceutical, Boehringer Ingelheim, Astellas, Daiichi-Sankyo, Win International, Japan Lifeline, and Nipro. The other authors have no conflicts of interest to disclose.
